# Calculation of the ELISA’s cut-off based on the change-point analysis method for detection of *Trypanosoma cruzi* infection in Bolivian dogs in the absence of controls

**DOI:** 10.1590/0074-02760160119

**Published:** 2016-07-04

**Authors:** Frédéric Lardeux, Gino Torrico, Claudia Aliaga

**Affiliations:** 1 Institut de Recherche pour le Développement, La Paz, Bolivia; 2 Instituto Nacional de Laboratorios de Salud, La Paz, Bolivia

**Keywords:** ELISA, cut-off, change-point analysis, Trypanosoma cruzi, dog, Bolivia

## Abstract

In ELISAs, sera of individuals infected by *Trypanosoma cruzi* show absorbance values above a cut-off value. The cut-off is generally computed by means of formulas that need absorbance readings of negative (and sometimes positive) controls, which are included in the titer plates amongst the unknown samples. When no controls are available, other techniques should be employed such as change-point analysis. The method was applied to Bolivian dog sera processed by ELISA to diagnose *T. cruzi* infection. In each titer plate, the change-point analysis estimated a step point which correctly discriminated among known positive and known negative sera, unlike some of the six usual cut-off formulas tested. To analyse the ELISAs results, the change-point method was as good as the usual cut-off formula of the form “mean + 3 standard deviation of negative controls”. Change-point analysis is therefore an efficient alternative method to analyse ELISA absorbance values when no controls are available.

In continuous diagnostic clinical tests, the establishment of a reliable cut-off is of paramount importance to discriminate between infected and non-infected individuals. Several standard methods have been proposed to choose optimal cut-offs (Lopez-Raton et al. 2014), and all require known positive and negative individuals to compute the cut-off value that will best discriminate. Enzyme-linked immunosorbent assay (ELISA) is a diagnostic tool carried out commonly in parasitological studies to detect antibodies or antigens related to a specific parasite. They produce absorbance readings, and to discriminate amongst positive and negative results, a cut-off value is needed. The determination of an optimal cut-off value in ELISA assays has long been a concern (Ridge & Vizard 1993). Generally, and especially with home-made ELISAs, cut-off values are estimated using known independent negative sera (sometimes along with positive ones) which are included in the titer-plates amongst the unknown samples. A general formula for a cut-off value is of the form:


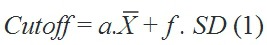


Where 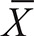 is the mean and *SD* the standard deviation of independent negative control readings, and *a* and *f* two multipliers.

Depending on authors, the multipliers can be set arbitrarily, for example to *f* = 0 with *a* = 2 or *a* = 3 (i.e., *cut-off* = twice or three times the mean absorbance obtained from the negative controls), or *a* = 1 with *f* = 3 (i.e., *cut-off* = mean + 3 times the standard deviation) (Classen et al. 1987). However, Frey et al. (1998) claimed that the cut-off can be statistically determined by setting *a* = 1 and *f* = *t*.√1 + (1/*j*) where *j* is the number of negative controls used in the plate and *t* is the (*1-α*)^th^ percentile of the one-tailed Student *t*-distribution with (*j-1*) degrees of freedom.

To detect infection by *Trypanosoma cruzi*, the causative agent of Chagas disease, Pan et al. (1992) have proposed another formula that takes into account negative and positive controls:


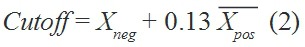


Where 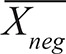 is the mean of the negative controls, and 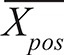 the mean of the positive controls.

When no controls are available, the above formulas cannot be used. Change-point analysis is a statistical analysis that can detect in a series of (ascending) values, a step indicating a change. Such change exists in a series of negative and positive ELISA values from a titer plate and should be detected with such an analysis.

The scope of the present study is to evaluate the change-point analysis as a tool to identify positive ELISA reactions when no controls are available. A set of dog sera from a field survey is used to diagnose *T. cruzi* infection and results are compared to those obtained using a standard approach using cut-off values from the usual equations (1) and (2).

## MATERIALS AND METHODS

*Negative dog sera* - Negative sera were from 16 dogs living in the city of La Paz, where no Chagas transmission exists. Dogs were born in the city and never went out in an endemic Chagas region. Negativity was checked by the Chagas STAT-PAK rapid test which is an accurate test for Chagas diagnosis in dogs (Nieto et al. 2009), and by polymerase chain reaction (PCR) targeting the kDNA of *T. cruzi* following Fernandes et al. (2001), slightly modified by one of us (Aliaga et al. 2011). The 16 negative sera were included as negative controls in each of the processed titer-plates.

*Positive dog sera* - 10 positive dog sera were obtained from dogs originated from the same region of the field sample (see below) and diagnosed positive both by PCR using the same protocol as above, and by the Chagas STAT-PAK rapid test following the manufacturer’s instructions. Then, in each ELISA plate, five-10 of them were included as positive controls to allow the computation of a cut-off value with formula *F*_*3*_ (Table I).

**TABLE I:** Cut-off formulas

Formula	*a*	*f*	Computation	Comment
*F*_*1*_	2	0	2 x MEAN of negative controls	-
*F*_*2*_	3	0	3 x MEAN of negative controls	-
*F*_*3*_	1	0	MEAN of negative controls + 0.13 x MEAN of positive controls	Pan et al. (1992) formula
*F*_*4*_^*a*^	1	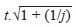	MEAN + *f* x SD, with *f* = 2.197	Frey et al. (1998) formula. Confidence level (1-α) for *t* computation: 97.5%
*F*_*5*_^*a*^	1	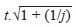	MEAN + *f* x SD, with *f* = 3.848	Frey et al. (1998) formula. Confidence level (1-α) for *t* computation: 99.9 %
*F*_*6*_	1	3	(MEAN + 3 x SD) of negative controls	Classen et al. (1987)

*a*: for the computation of *F*_*4*_ and *F*_*5*,_ *j* is the number of negative controls used in the plate (16 in the present study) and *t* is the (*1-α*)^th^ percentile of the one-tailed Student *t*-distribution with (*j-1*) degrees of freedom. Because 16 negative controls were used in the study, and taking into account the confidence level for the computation of the Student *t*, the *f* values were 2.197 and 3.848 for *F*_*4*_ and *F*_*5*_ respectively.

*Sera of field sample* - A field sample of 231 dog sera was obtained from four Bolivian populations. Villages of dog’s origin were Eje Pampa (Lat -18.54º Long -65.17º) (47 individuals) and Lagar Pampa (Lat -18.45º Long -64.99º) (26 individuals) in the dry inter-Andean valleys, and La Brecha (Lat -19.51º Long -62.56º) (72 individuals) and Palmarito (Lat -19.49º Long -63.46º) (78 individuals) in the Chaco region. For each dog, 10 mL of blood was taken from the cephalic vein. 5 mL were put in 6 M Guanidine Hydrochlorid/EDTA 0.2 M for DNA analysis (for *T. cruzi* identification) and 5 mL in EDTA vacuum tubes for the ELISAs. At the field site, blood samples were allowed to clot and were kept at 4ºC.

*IgG-ELISA protocol to detect antibodies against T. cruzi* - In the laboratory, tubes containing blood samples of dogs were centrifuged at 3000 rpm for 10 min for plasma separation. The ELISA protocol was from Lauricella et al. (1998) which is routinely used for Chagas diagnosis in dogs (Enriquez et al. 2013). It was slightly modified as follow: ELISAs were carried out in 96-well micro-titer plates (NUNC Maxisorp, flat bottom) coated with a homogenate of *T. cruzi* epimastigote culture. The homogenate was prepared as follow: 1 mL of pure culture of epimastigotes (forms cultured at 28ºC in LIT liquid medium) was centrifuged in a 5 mL Eppendorff tube at 4000 rpm at 4ºC for 10 min. The supernatant was discarded, 1 mL of phosphate buffer saline (PBS) at pH7.2 was added and the tube vortexed. This washing operation was realised three times. Then, 1 mL of PBS was added and the tube vortexed. A dilution of 1/1000 of the solution was realised in PBS in carbonate buffer (100 µL of “parasites” in PBS + 9900 µL of carbonate buffer), vortexed, and 100 µL of the solution was then added in each well. The plate was sealed with adhesive plastic sheet and incubated overnight at 4ºC. The following day, the content was discarded by inversion. The plate was washed three times with 120 µL/well of washing buffer (PBS - 0.01% Tween 20). Then each well was loaded with 100 µL of blocking buffer (PBS - 3% skimmed milk REGILAIT, France) and incubated 1 h at 37ºC. Then, the plate was washed three times with 120 µL/well of washing buffer. Dog sera were diluted at 1/100 in dilution buffer (PBS - 1% skimmed milk) in 1.5 mL Eppendorff tubes, vortexed and kept at 4ºC until loaded in the plate. Diluted sera were loaded in duplicate at 50 µL/well and incubated 1 h at 37ºC. The plate was then emptied by inversion and washed three times with 120 µL/well of washing buffer. Anti-dog IgG were diluted at 1/1200 in dilution buffer. Each well was loaded with 50 µL of peroxidase conjugated antibodies anti-IgG and incubated 1 h at 37ºC. Then the plate was emptied by inversion and washed three times with 120 µL/well of washing buffer. Then 50 µL of TMB (3, 3’, 5, 5’ - Tetramethylbenzidine, SIGMA) was added in each well and the plate was incubated for 5 min at room temperature. Then, 50 μL/well of sulfuric acid 1 N were added to stop the reaction and absorbance values were obtained at 450 nm in a microwell plate reader (Multiskan). The mean absorbance of each pair of duplicate sera was calculated. When the difference between both values was more than 30%, the sample was retested (Lauricella et al. 1998). In total, the 231 dog sera and the controls were processed in seven titer plates.

*Cut-off formulas* (Table I) - For each of the seven titer-plates analysed, cut-off values were computed using six usual formulas (*F*_*i*_, *i* = 1 to 6). The value of the *f* coefficient in formulas *F4* and *F5* was 2.197 and 3.848 respectively, according to Frey et al. (1998).

*Change-point analysis* - The whole set of sera was also analysed by change-point analysis which does not need the presence of known positive or negative sera (blind analysis). Change-point analysis is aimed at identifying points in a series where the statistical properties change. In particular, such analysis can be used to detect abrupt steps in the mean level of a series. In the case of ELISA, if absorbance values of a micro-titer plate are ordered in ascending order, negative samples are supposed to be the lower ones in the series while positive ones (if they exist) would be the higher. However, values are not supposed to increase regularly if positive samples exist in the series. Indeed, as positive controls are supposed to be “different” from negative ones, a step, even small, should appear in the series, separating the negative from the positive values. Therefore, change-point algorithms might be used to detect such a change and locate the value where in the series this change occurs. The detected value is therefore a kind of specific cut-off proxy that discriminates between positive and negative samples.

For each of the seven processed titer-plates, absorbance values were first arranged in ascending order and each series was analysed using the R package “changepoint” (Killick & Eckley 2014) which detects a change-point if it exists and locates it in the series. In this package, the Pruned Exact Linear Time (PELT) algorithm was selected (Killick et al. 2012) with the CUSUM method as detection option (Page 1954). The PELT algorithm divides iteratively the series of absorbance values in sub-groups of increasing size. In each, it calculates the minimum of a “cost” function that takes into account the method of detection (Killick et al. 2012). Minima indicate where the change-points are located within the series. The PELT algorithm can therefore rapidly detect various change-points in a series. The CUSUM method is based on cumulative sums and operates as follow: The absorbance values *x* are ordered in ascending values (*x*_*1*_ *,…x*_*n*_) and sums (*S*) are computed sequentially as *S*_*0*_ = 0, *S*_*i+1*_ = max (0, *S*_*i*_ *+ x*_*i*_ - *L*_*i*_), where *L*_*i*_ is the likelihood function. When the value of *S* exceeds a threshold, a change-point has been detected.

Approval for the study was granted by the WHO’s Research Ethics Review Committee (ERC), project #A90281 and by the Comisión de Ética de la Investigación del Comité Nacional de Bioética (CEI-CNB) of Bolivia (letters 3 august 2010 and 21 august 2012).

## RESULTS

In each plate, the change-point analysis identified only one change-point, and each time, it correctly discriminated between positive and negative controls. No positive control was classified as negative and no negative control was classified as positive. The change-point analysis identified step point values which, unlike formulas *F*_*1*_, *F*_*5*_ or *F*_*6*_, pose no false negative identification problems.

A correct cut-off value should lie, at least, between the highest value of the negative controls and the lowest value of the positive controls. In that sense, only the formulas *F*_*1*_ (i.e., 2 x MEAN of negatives), *F*_*5*_ and *F*_*6*_ (*F* = MEAN + *f*. SD of negative controls, with *f* ≥ 3) correctly discriminated amongst the positive and the negative controls. All other formulas failed in some instances, giving in some plates cut-off values sometimes below the highest known negative control values (*F*_*3*_ or *F*_*4*_), or above the lowest positive control values (*F*_*2*_) (Table II). A sub-estimation or an over-estimation of the cut-off value leads to the determination of false positive or false negative results respectively. For example, in plate 3, formula *F*_*2*_ overestimated the cut-off value. Five positive controls were therefore erroneously classified as negative and when the whole sample of 231 dogs was considered, eight unknown dogs mixed among the positive controls (which should therefore be identified as positive), were erroneously classified as negative. On the contrary, some negative reactions might be erroneously classified as positive as occurs with *F*_*4.*_ Indeed, across the seven titer plates, 12 true negative reactions out of 112 (= 7 plates x 16 negative controls) were erroneously classified as positive, and from the unknown sera, 53 out of the 231 field dogs would be identified as positive when only 34 were classified positive with the formula *F*_*1*_. Formulas *F*_*1*_, *F*_*5*_ and *F*_*6*_ that correctly separated positive from negative controls, gave low cut-off values, increasing the risk of detecting false positives as it might be the case in plates 4, 5 and 6. Caution should therefore be taken when using *F*_*1*_, *F*_*5*_ or *F*_*6*_.

**TABLE II:** Absorbance cut-off values for each of the seven titer plates, computed with formulas *F_1_ - F_6_* and step points detected by the change-point analysis

Plate	F_1_	F_2_	F_3_	F_4_	F_5_	F_6_	Change-point analysis	Highest negative control	Lowest positive control
1	0.519	0.779	0.418	0.441	0.578	0.508	0.471	0.471	0.862
2	0.494	0.741	0.404	0.369	0.461	0.414	0.710	0.380	0.710
3	0.614	0.920	0.473	0.455	0.566	0.509	0.462	0.462	0.941
4	0.503	0.755	0.414	0.374	0.466	0.419	0.496	0.370	0.808
5	0.424	0.636	0.358	0.359	0.469	0.412	0.598	0.343	0.598
6	0.456	0.684	0.384	0.354	0.448	0.400	0.570	0.356	0.843
7	0.552	0.828	0.475	0.415	0.519	0.466	0.432	0.420	1.115

## DISCUSSION

Unlike the usual cut-off formulas, the change-point analysis does not need the presence of controls, either negatives or positives in the ELISA titer plate to compute a cut-off value. When the series of absorbance values is arranged in ascending order, the algorithm is intended to find a step point from which the statistics in the series change (i.e., a change from negative to positive reactions). In the present study, the change-point analysis divided each titer plate into two subsamples of negative and positive reactions, and did not misclassify known positive or negative samples as it happened with formulas *F*_*2*_, *F*_*3*_ or *F*_*4*_. Therefore, the method appears to be a method of choice when no controls are available.

From the field study, formulas *F*_*4*_ (and to a lesser extend *F*_*3*_) were likely to overestimate the number of positive samples, and *F*_*2*_ to underestimate them. They failed in some plates to correctly identify known positive or negative controls and therefore cannot be recommended. Cut-off values computed from formulas *F*_*1*_, *F*_*5*_ or *F*_*6*_ are likely to better separate positive from negative samples. However, all of these might compute cut-off estimates slightly too low, giving some false positive results. Cut-off values from formula *F*_*6*_ (i.e. with *f* = 3) lie between those estimated with *F*_*4*_ and *F*_*5*_. Indeed, because 16 negative controls are used and depending on the confidence level, *f* is almost < 3 in *F*_*4*_ and almost > 3 in *F*_*5*_. With six independent negative controls, *f* would be 2.777 at the 97.5% confidence level and 6.366 at the 99.9% confidence level. The present results indicate that a *f* multiplier of at least 3 (as in *F*_*6*_) should be recommended.

The detection of true positive or true negative individuals is always difficult when individual absorbance values are close to the cut-off value. For that reason, as far as Chagas disease is concerned, the precise detection of cases is usually carried out using several independent assays. For example, ELISA and indirect immunofluorescence assay (IFA) are first carried out, and if the results are not in agreement, a third assay is carried out [r-ELISA (recombinant ELISA) for example]. Although the change-point analysis may detect small changes in a series and has correctly discriminated between the known positive and negative samples of the study, it does not solve the sensitivity problem. False negative or false positive results can therefore exist (as small proportions however). As in a standard procedure, it can be recommended to re-test the samples with other independent assays, in particular those for which the absorbance value lies close to the detected change-point value.
